# Dynamics of picosecond laser ablation for surgical treatment of colorectal cancer

**DOI:** 10.1038/s41598-020-73349-w

**Published:** 2020-11-20

**Authors:** R. J. Beck, I. Bitharas, D. P. Hand, T. Maisey, A. J. Moore, M. Shires, R. R. Thomson, N. P. West, D. G. Jayne, J. D. Shephard

**Affiliations:** 1 Institute of Photonics and Quantum Sciences, Heriot-Watt University, Edinburgh, EH14 4AS UK; 2 Leeds Institute of Medical Research At St. James’s, University of Leeds, Leeds, LS9 7TF UK

**Keywords:** Biophysics, Cancer, Medical research, Physics

## Abstract

Endoluminal surgery for the treatment of colorectal neoplasia is typically carried out using electrocautery tools which imply limited precision and the risk of harm through collateral thermal damage to the adjacent healthy tissue. As a potential alternative, we present the successful colonic epithelial laser ablation by means of picosecond laser pulses. Laser ablation studies performed in ex-vivo colon tissue result in cavities with comparable thickness to early stage colorectal cancers. The corresponding histology sections exhibit only minimal collateral damage to the surrounding tissue and the depth of the ablation can be controlled precisely by means of the pulse energy. High-speed imaging has been used for the first time to visualize picosecond laser ablation of cancerous tissue in a clinically relevant model. This information was correlated with histopathology and optical surface profilometry revealing the dynamic nature of the laser tissue interaction and the need for temporal or spatial separation of pulses for optimum efficacy with regards to tissue removal. Overall, the application of picosecond laser pulses to ablate endoluminal bowel lesions demonstrates significantly improved precision and reduced thermal damage to the adjacent tissue in comparison to conventional procedures and hence will enable more precise surgical treatment of cancers.

## Introduction

Colorectal cancer is a leading cause of cancer and cancer-related mortality worldwide^1^. Every year, there are approximately 300,000 new cases and 200,000 deaths in Europe and the USA. As a consequence of modern cancer screening, colorectal cancers are more likely to be detected at an early or premalignant disease stage. Until now, endoluminal surgery for the treatment of early colorectal neoplasms (adenomas and malignant polyps) is typically based on conventional diathermic techniques which imply restrictions on precision and the risk of harm through collateral thermal damage to the surrounding healthy tissue, resulting in bowel perforation. As a potential alternative to mitigate these risks, we present the successful laser ablation of colorectal polyps and healthy intestine with high accuracy and only very minimal impact on the adjacent tissue by means of picosecond pulsed lasers. The application of lasers in medicine is a rapidly growing field and a variety of surgical laser treatments are well established, amongst others in ophthalmology^2^, dermatology^3^ and the ear, nose and throat (ENT) area^4,5^. Clinically established laser-based surgical procedures typically utilize continuous wave (cw), longer pulsed or nanosecond pulsed laser sources where the laser-tissue interaction is based on linear absorption and thermal ablation^4,5^. Thermal diffusion results in collateral damage to adjacent tissue regions and severely limits the precision of the tissue ablation.

In contrast, ultrafast or ultrashort laser pulses with their very high peak intensities offer the distinct advantage of non-linear absorption processes and enable a predominately non-thermal ablation regime resulting in a significantly improved surgical precision. Laser pulse lengths of 10 ps and shorter are typically considered as ultrashort pulses^6^. As a consequence of the non-linear absorption of the focused ultrashort laser pulse a free electron plasma is generated. This results in a laser-induced optical breakdown followed by an energy transfer leading to the formation of a shock wave and a cavitation bubble. An in-depth discussion of the underlying complex laser-tissue interaction for ultrashort pulsed lasers and the material removal mechanisms including the dynamics of the cavitation bubbles can be found in *Vogel *et al*.*^7,8^, *Tinne *et al*.*^9^ and *Linz *et al*.*^10^. The application of ultrashort laser pulses has been suggested for various clinical indications, in particularly in ophthalmology^11^, but also in dentistry for the ablation of enamel and dentin^12^, in ENT for vocal fold surgery^13^ and cardiology for the removal of atherosclerotic plaque^14^.

Kerse et al.^15^ presented the so-called ablation-cooled material removal using bursts of femtosecond laser pulses. In this case, successive laser pulses enable to remove the residual heat accumulation within the target material by ablating the material prior to heat diffusion. This improves the efficiency of the material removal by a factor of 10 compared to previous work on metals^16,17^. The reported tissue removal rate for *ex-vivo* brain tissue is 2 mm^3^/min.

With regards to a practical application of ultrashort pulses surgery for endoluminal surgery and the ablation of colonic polyps and early stage cancers however, a much higher material removal rate would be required to fulfil the clinical requirements in terms of effectiveness and cost-efficiency. In this study, we developed a picosecond laser pulsed process for effective tissue ablation with only minimal collateral damage. The impact of the process parameters on the tissue removal rate and the thermal damage for ex-vivo tissue models will be discussed. In this work, high-speed imaging has been used for the first time to visualize picosecond laser ablation of neoplastic tissue in a clinically relevant model. Such imaging provides critical information on the process dynamics which can then be correlated to histopathological analysis and optical surface profilometry of the ablated cavities enabling parameter optimization.

## Materials and methods

### Experimental setup

Two commercially available picosecond laser workstations were used for the laser ablation experiments, both with a pulse duration of 6 ps. The Trumpf TruMicro 5x50 laser has a wavelength of 1030 nm and a maximum pulse energy of 125 µJ. The Light Conversion Carbide operates at a wavelength of 1028 nm and provides a pulse energy of up to 85 µJ. The laser beam is for both systems focused onto the tissue surface and scanned across its surface by means of a galvanometer scanhead. The laser workstations use f-theta lenses with different focal lengths of 160 mm (TruMicro) and 100 mm (Carbide), resulting in a focussed spot diameter of 30 µm and 20 µm respectively. The position of the top surface of the tissue relative to the laser focus is determined using a microscope system to account for the varying thickness of the tissue samples. Whilst this work focusses on the optimisation of the laser tissue-interaction and tissue removal, a fibre delivery of the used laser pulses is feasible with regards to an endoscopic application^18,19^. The process could be deployed through the working channel of a standard colonoscope. To further investigate the dynamics of the laser tissue interaction a high-speed camera, Photron Fastcam Mini AX200, has been used. This monochrome camera has a resolution of 1280 × 616 pixels at a frame rate of 10 kHz^20^. The camera was fitted with a 7:1 zoom lens resulting in viewing field of approximately 1.6 × 1.2 mm at a working distance of 135 mm.

### Tissue models

**Figure 1:**
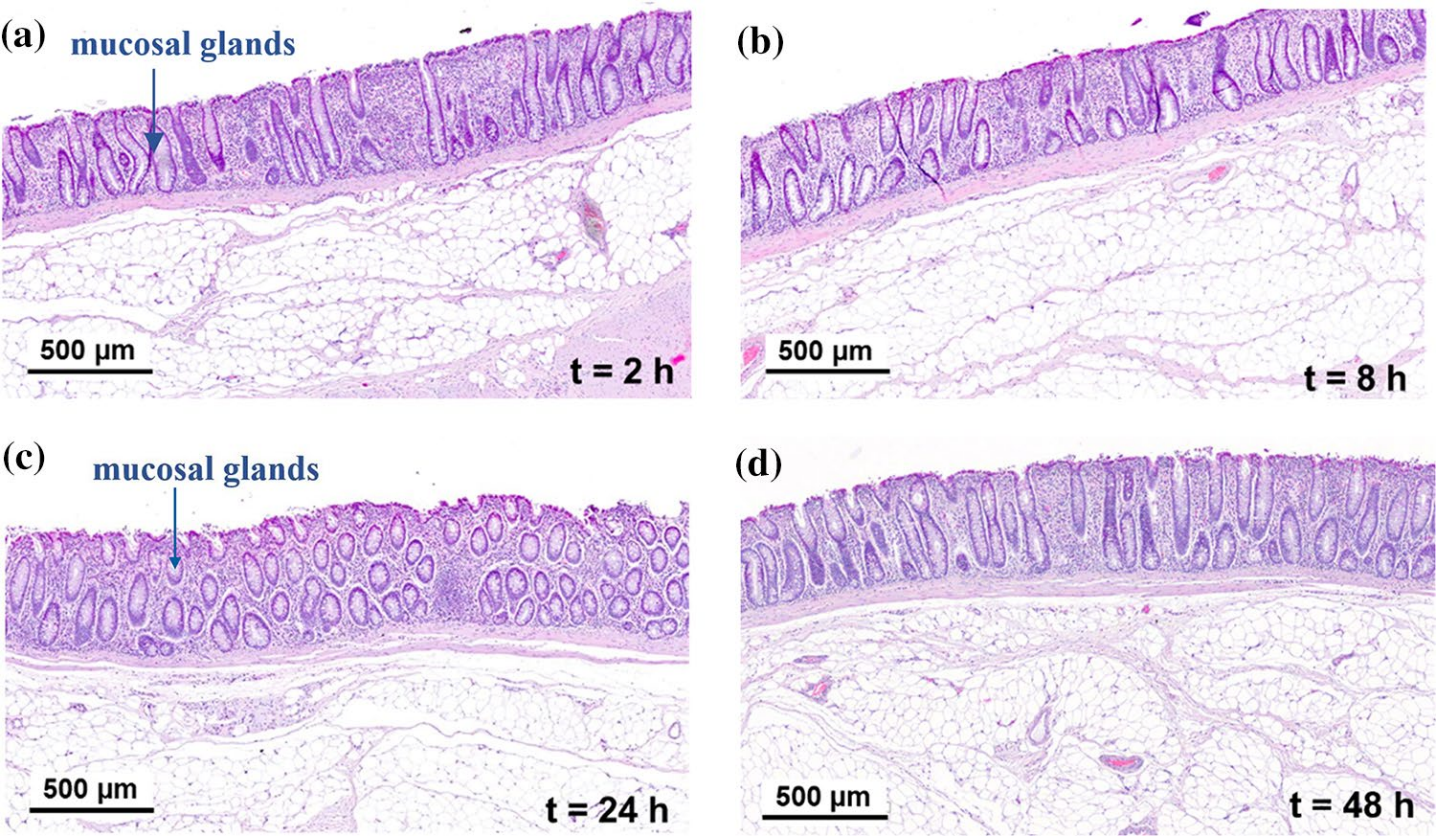
Validation of stable tissue morphology: routine histology sections of pig intestine mucosa and submucosa at various times after tissue extraction when stored in PBS on ice.

This work is based on fresh *ex-vivo* tissue using two different, clinically relevant animal models: healthy pig intestine and an established mouse model of colorectal neoplasia. Porcine tissue is a well-established model to study gastrointestinal diseases and colonic tissue due to the physiological similarities with humans^21^. Human and porcine intestine exhibit a very similar tissue morphology and similar dimensions. However, pigs do not express cancerous lesions in the colon and hence an Apc(Min/ +) mouse model as a popular animal model for human colon neoplasia^22,23^ was also used. In this case, the development of multiple polyps in the colon is caused by a mutation of the Apc gene. Animals were handled in the study and all methods were carried out in accordance with relevant guidelines and regulations. All experimental protocols were approved by the UK NHS Health Research Authority. The project license number under which this tissue model was developed and supplied is 707,965. Ethics approval for the research has been granted by the Ethics Committee at Heriot-Watt University. The tissue samples are stored in phosphate-buffered saline (PBS) solution on ice between the sample extraction and the experiments. The histology is based on formalin fixation, routine processing into paraffin wax and staining using haematoxylin and eosin. Additional tissue samples were prepared and fixed in Glutaraldehyde after the laser ablation for surface profilometry in order to access the tissue removal rate and the uniformity of the rablated cavities. The tissue samples remained fresh and morphologically stable for a time period up to 48 h without noticeable tissue degradation (see Fig. 1) when stored in PBS on ice. It should be highlighted that the apparent different shape of the mucosal crypts in Fig. 1c compared to 1a, b and d is caused by cross-sectioning of the tubular-shaped structures.

## Results

### Laser ablation at low repetition rates

**Figure 2:**
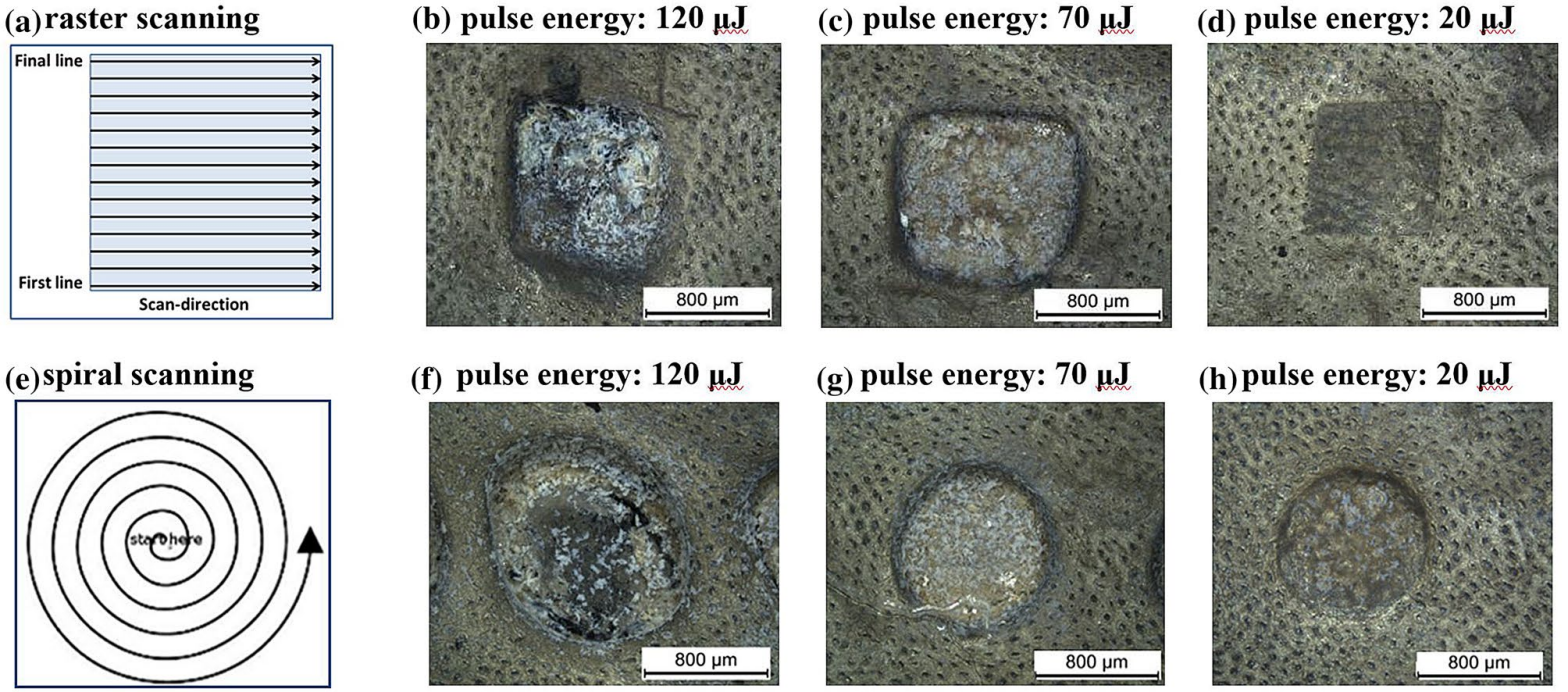
Picosecond laser ablation of pig intestine at a repetition rate of 1 kHz: (**a**) scanning pattern for raster scanning, (**b**–**d**) micrographs for different pulse energies. (**e**–**g**) corresponding scanning pattern and micrographs for spiral scanning.

For the optimisation of the laser parameter with regards to an effective tissue ablation, linear raster scans were carried out (see Fig. 2a) with different laser repetition rates, pulse energies and separations between individual laser spots. The focal diameter of the laser beam on the tissue was 30 μm and the laser repetition rate was set to 1 kHz. In order to achieve a spot separation of 10% between successive laser pulses, the laser beam was scanned at a speed of 3 mm/s across the tissue surface. The individual lines of the raster scanning patter were separated by 3 μm for a 10% separation laterally. As a result, the ablation of well-defined square cavities was achieved for pulse energies of 120 μJ and 70 μJ (Fig. 2b,c). Figure 2f–h show micrographs for the laser ablation using an outward spiral scanning pattern. An Archimedean spiral (Fig. 2e) is preferable to the square raster scan as it enables a continuous emission of the laser pulses and thus avoids the requirement of turning the laser emission off during the fly-back times for the raster scanning. Outward spiral scans are better suited for tissue ablation, since inward spirals may result in unsupported cylindrical tissue structures during the scan. As before, a scanning speed resulting in a spot separation of 10% between successive laser pulse and a separation of 10%, i.e. 3 μm, between the individual turnings of the spiral was found to result in clear and well-defined circular cavities. For laser fluences below the plasma threshold (i.e. Figure 2d,h), the laser has only a very superficial impact on the tissue.

**Figure 3:**
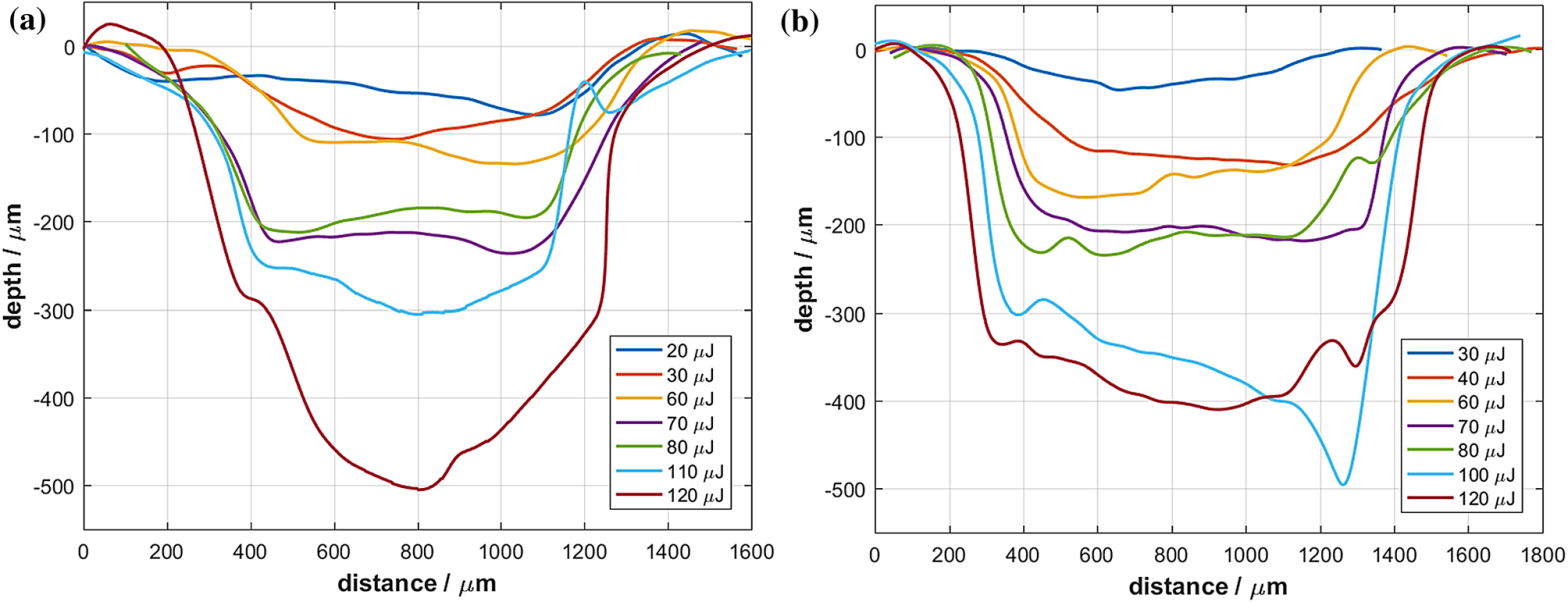
Cross-sections through middle of ablated cavities for different pulse energies: (**a**) for raster scanning and (**b**) for spiral scanning.

**Figure 4:**
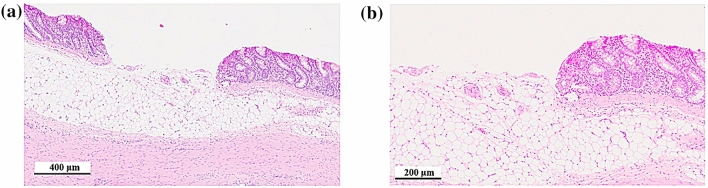
Histology section of laser resected cavity for spiral scanning at a repetition rate of 1 kHz and a pulse energy of 80 µJ at different magnifications.

Figure 3 shows the horizontal cross-sections through the middle of the ablated cavities for both scanning patterns for different pulse energies. The depth of the ablation can be controlled by means of the pulse energy. With regards to the practical application in endoluminal surgery, this is an important feature to avoid bowel perforation as a serious complication. Areas showing deeper invasion of neoplastic tissue could be ablated sequentially using multiple laser scans. Small variations in the ablation depth between Fig. 3a,b are caused by morphological differences between the samples. The corresponding histology section for spiral scanning at a pulse energy of 80 µJ (see Fig. 4) exhibits only minimal collateral thermal damage, i.e. the thickness of the necrosis tissue is smaller than 50 μm. The mucosal glands close to the interface of the laser ablated region are unaffected. The tissue removal rate at a pulse energy of 80 µJ was 0.4 mm^3^/min.

### Laser ablation at higher repetition rate

**Figure 5:**
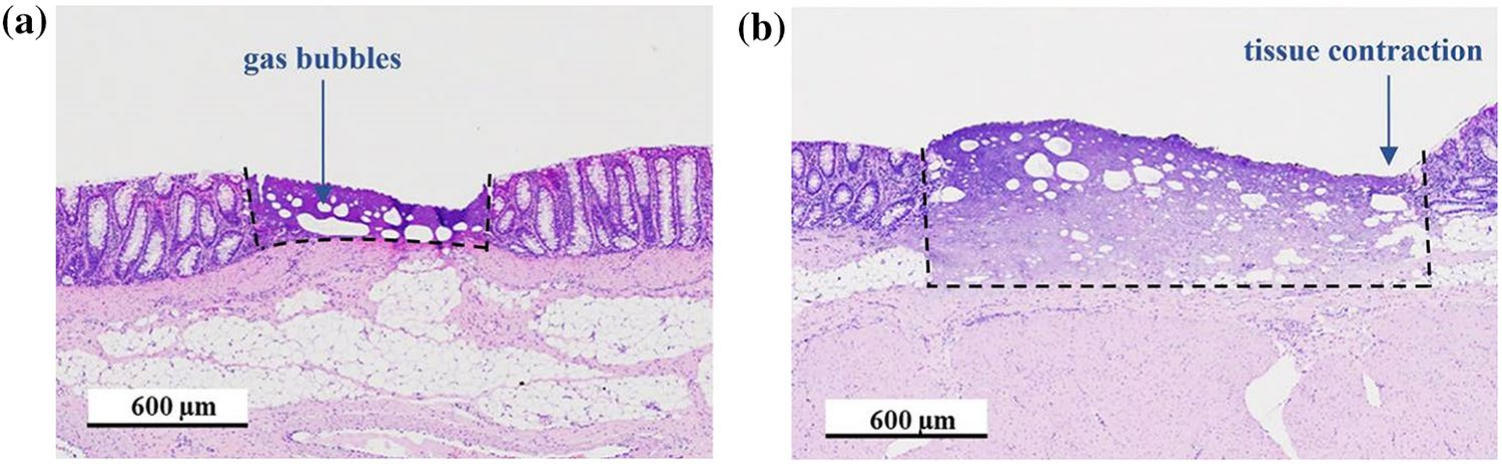
Histology sections of pig intestine after laser ablation at a repetition rate of 10 kHz and a 10% spot separation with significant thermal coagulation as a result of the cavitation effect: pulse energy for (**a**) 60 µJ and (**b**) 80 µJ. The boundaries between the coagulated and unaffected tissue regions are highlighted with dashed lines.

With regards to the practical application of this process in a clinical environment, the overall time to ablate early stage cancerous lesions is important. By increasing the laser repetition rate, and consequently the rate of energy deposition, the tissue removal rate can be further increased. However, at laser repetition rates typically above 3 to 5 kHz the dynamics of the plasma mediated ablation process and in particular the cavitation bubbles become more critical. The temporal and spatial separation of the individual laser pulses are crucial for the efficacy of the tissue removal. Furthermore, this also depends on the tissue morphology and the mechanical properties of the individual layers. Figure 5 shows histology sections for the laser ablation at 10 kHz repetition rate and pulse energies of 60 and 80 μJ for a pulse separation of 10%, i.e. a spatial separation of 3 μm between successive laser pulses. The scan speed of the laser was 30 mm/s for a spot diameter of 30 μm. As a consequence of the low spatial separation of the laser pulses, there is a very strong interaction of the individual cavitation bubbles resulting in tissue coagulation. This results in thermal damage that is similar to the outcome when using conventional diathermy instruments and the tissue contraction as seen on the right part of the coagulated region is comparable to scarring. However, it should be highlighted that for the case of the picosecond laser the margins between coagulated and unaffected tissue are more well-defined in comparison to those with an electrocautery device. Also, gas bubbles caused by the cavitation effect are only visible for laser ablation and are not present when using diathermy. Within these coagulated tissue regions the cell nuclei are fully denatured and hence this could be a viable method to treat early stage cancerous lesions. Furthermore, a deliberate heat deposition when using the picosecond laser, as a consequence of this cavitation effect, could potentially be useful for on-demand haemostasis and laser coagulation.

**Figure 6:**
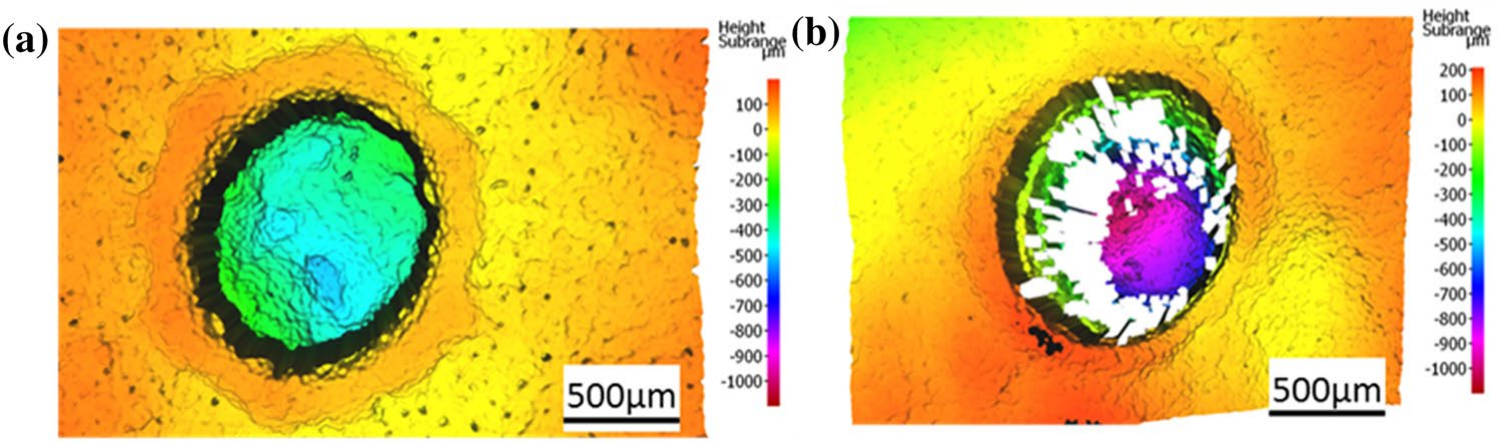
Surface profiles of laser ablation at a repetition rate of 20 kHz using four spiral scans at a spot separation of 100% and pulse energies of (**a**) 66 µJ and (**b**) 85 µJ. The white areas in b) are missing data points due to the high gradient of the ablated cavity.

**Figure 7:**
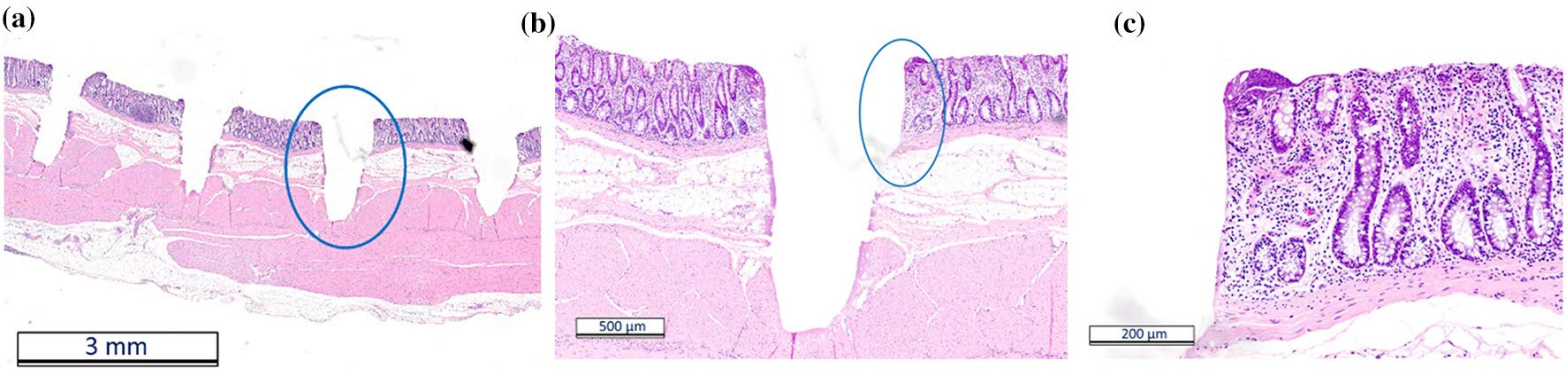
Histology sections of pig intestine for laser ablation at a repetition rate of 20 kHz and a spot separation of 100%. (**b**,**c**) show the zoomed-in regions of the regions marked with a blue ellipse of each previous cross-section.

From these results it was proposed that an increase of either the spatial or the temporal separation between successive laser pulses is necessary to overcome the adverse impact of cavitation. By significantly increasing the spot separation between successive pulses interaction with the resulting cavitation bubbles can be avoided^9^. According to Vogel et al*.*^24^, the diameter of the cavitation bubble is dependent of the pulse energy of the laser or respectively the energy density. The laser pulse energies used in these experiments were up to 85 µJ and the diameter of the focused laser spot was 20 µm. It was found that under these conditions a pulse separation of 100% of the spot diameter was sufficient to prevent the adverse impact of cavitation and to enable an effective laser ablation. The distance of the individual turns of the spiral pattern is less critical for the outcome of the laser ablation. A distance of 4 µm for the turns of a focused spot diameter of 20 µm, corresponding to a 20% separation laterally, resulted in the ablation of repeatable and well-defined circular cavities. For a better comparison with the results for low pulse repetition rates and high spot overlap as presented above, each spiral scan has been repeated 4 times to achieve a better match of the total deposited laser energy. Figure 6 shows laser ablated cavities for 4 repetitions of spiral scans at a repetition rate of 20 kHz and a spot separation of 100% (scanning speed = 400 mm/s). The depth of the ablation can be controlled again by means of the pulse energy and the corresponding histology sections (Fig. 7) exhibit minimal collateral damage, i.e. smaller than 50 µm. For parts of the ablated cavity the cells were completely unaffected and viable even at the interface (Fig. 7c). The tissue removal rate for a laser pulse energy of 85 µJ was 6.3 mm^3^/min.

### Demonstration of high-repetition rate laser ablation on cancerous mouse intestine

**Figure 8:**
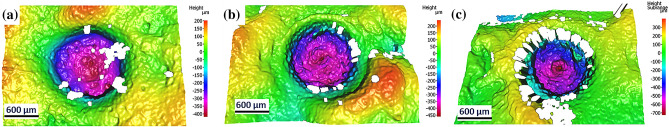
Surface profiles of laser ablated cavities in cancerous mouse intestine after 4 repetitions of a spiral pattern at a repetition rate of 20 kHz and pulse energies of (**a**) 30 µJ, (**b**) 44 µJ and (**c**) 60 µJ. The white areas are missing data points due to the high gradient of the ablated cavity.

**Figure 9:**
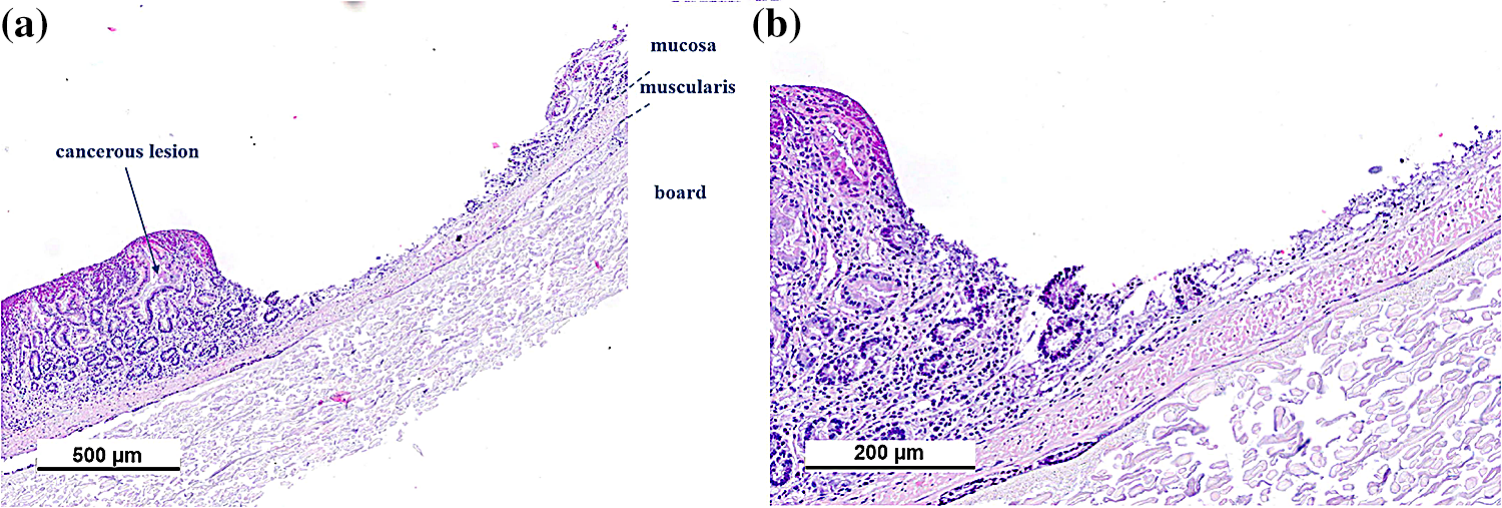
Histology section of laser ablated cavity of neoplastic mouse intestine with only minimal collateral damage. The bottom layer is a Dispocut board as substrate to support the delicate tissue. (**b**) is magnified view of (**a**).

The optimised processing parameters established on the porcine tissue model were subsequently translated to the mouse intestinal neoplasia model. Due to the tissue samples being thin (typically 500 microns) a layer of board (Dispocut) was used to support the tissue structures during the laser ablation and also during the tissue fixation and analysis. Scanning was carried out using a repetition rate of 20 kHz in an outward spiral pattern with a 4 µm hatch and a spot separation of 100% of the beam diameter between successive laser pulses. This resulted in the ablation of well-defined circular cavities without any noticeable cavitation effect. As before with the porcine model, the overall depth of the ablation can be controlled by means of the pulse energy as measured using optical surface profilometry (Fig. 8). By using a repetition of 4 spiral laser scans the total depth of the ablated cavities ranged between 300 und 700 µm depending on the size of the cancerous lesion without any perforation of the delicate tissue. The corresponding histology section for a pulse energy of 42 µJ and 4 spiral laser scans is shown in Fig. 9. A ~ 400 µm thick layer of neoplastic mucosa has been ablated without any impact on the muscularis mucosae and only marginal collateral thermal damage.

## Discussion

The successful colonic epithelial laser ablation by means of picosecond laser pulses is presented as a potential alternative to overcome limitations of conventional electrocautery tools in terms of precision and thermal damage for the surgical ablation of tumours. The results presented show that ultrashort laser pulses enable a well-defined mucosal ablation of cavities with comparable thickness to early stage colorectal cancer. For low laser repetition rates, i.e. below 5 kHz, a high spatial overlap, typically 90% of the spot diameter, between successive laser pulses enables tissue ablation with minimal collateral damage, i.e. < 50 µm. The depth of the laser ablation can be controlled by means of the pulse energy and thicker tissue regions can be removed sequentially using multiple scans. This is an important feature when considering clinical application by potentially eliminating the serious surgical complications such as bowel perforation.

At higher pulse repetition rates, the dynamics of the laser-tissue interaction, the plasma-mediated ablation process and, in particular, the interaction of cavitation bubbles with individual laser pulses become much more critical for the tissue ablation^8,9^. A low spatial separation of successive laser pulses, i.e. 10% of the focused spot diameter, and as a consequence a strong interaction between the cavitation bubbles generated by each pulse, results in a significant heat deposition into the tissue and an effective tissue coagulation (Fig. 5). However, it should be noted, that this would be a valid modality to treat early stage cancerous lesions since the cell nuclei get fully denatured by the laser energy. With these parameters residual gas bubbles remain visible in the histology cross sections. As a potential advantage, in comparison to conventional diathermy instruments, the margins between coagulated and unaffected tissue regions are more clearly defined and the depth of the coagulated region can be controlled by the pulse energy.

Nevertheless, what is more significant for the application of picosecond laser pulses in a clinical environment is the ability to ablate the tissue. A spot separation of 100% of the spot diameter mitigates these adverse effects of cavitation bubbles and the shock waves on the laser ablation. The position of the laser focus relative to the tissue surface is important for the quality of the laser ablation. It was found that focusing the laser in the region between the tissue surface and a maximum of ~ 400 µm above the surface resulted in best ablation results in terms of precision and minimal collateral damage, typically < 50 µm. This effective mucosal ablation was successfully demonstrated for both healthy pig intestine and cancerous mouse intestine.

**Figure 10:**
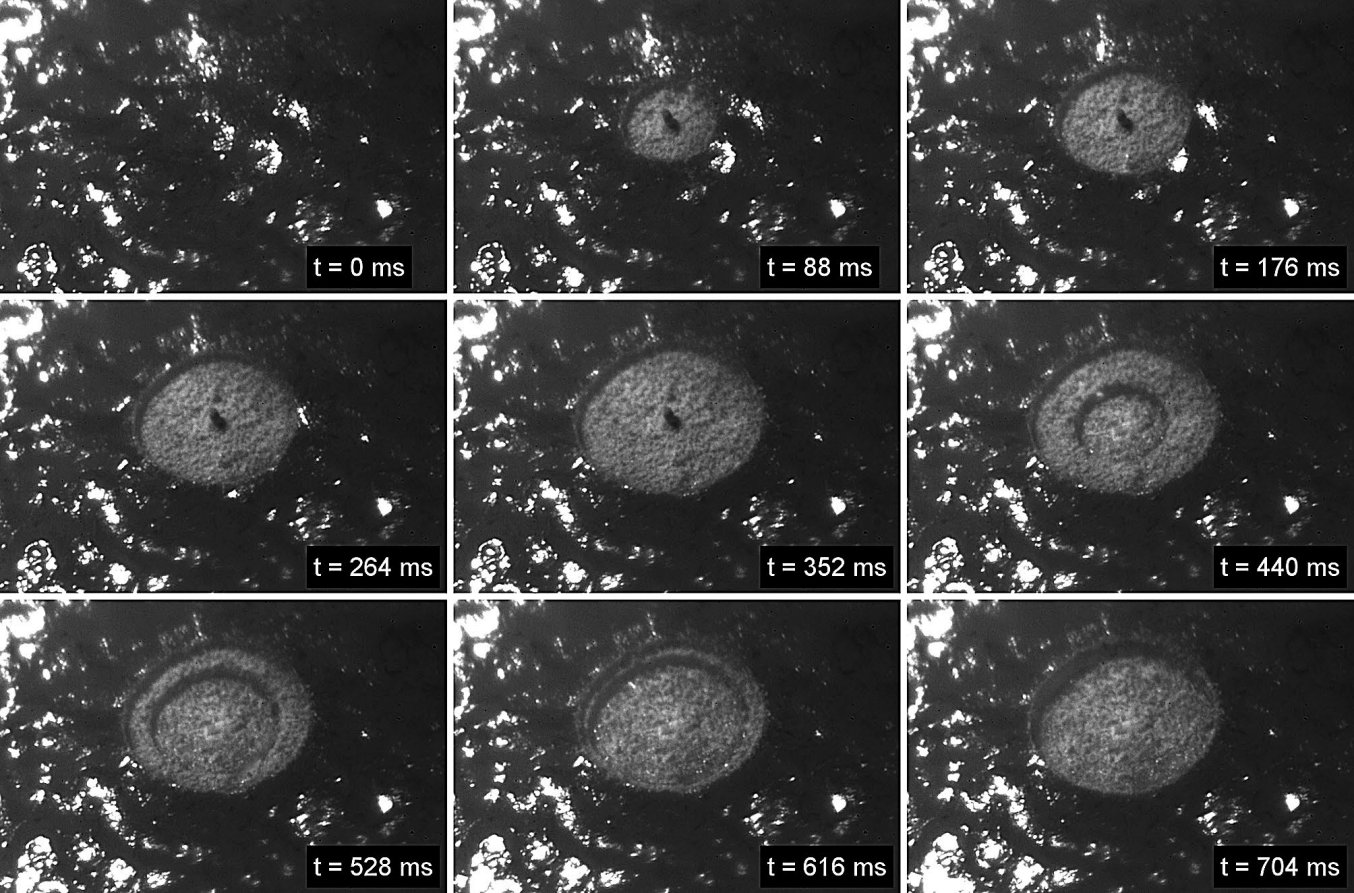
High speed footage of laser ablation of cancerous mouse intestine at a repetition rate of 20 kHz and a spot separation of 100% between successive laser pulses. The diameter of the ablated region is 600 µm.

To further investigate and visualize the dynamics of the laser tissue interaction, a high-speed camera (Photron Fastcam Mini AX200) has been used. This monochrome camera operating at a frame rate of 10 kHz enabled recording of the outcome of the laser ablation after every second laser pulse for a laser repetition rate of 20 kHz. The corresponding high speed footage (see Fig. 10 and supporting material 1) illustrates the tissue removal of neoplastic mouse intestine with high precision despite the non-uniform tissue morphology and surface structure of the neoplastic lesions (surface roughness ~ 20 µm). Each iteration of the spiral laser scan at a pulse energy of 42 µJ and a 100% spot separation enables removal of a tissue layer to a depth of ~ 60 µm. At the beginning of the first scan, there is a small volume of tissue coagulation, which is removed on the subsequent iteration. This is caused by heat accumulation at the center of the spiral and can be mitigated by increasing the internal diameter of the spiral from 20 µm to 30 µm. Importantly, the high speed video (supporting material 1) clearly visualizes the laser-tissue interaction and shows a clean and well-defined tissue removal process. Detailed analysis on a frame-by-frame basis indicates that there is no observable ejection of tissue that might consist of live cells. This gives confidence that there would be no risk of seed-infecting other parts of the colon. To our knowledge this is the first time such in-process visualization of neoplastic tissue ablation using a picosecond laser has been carried out on a clinically relevant model.

**Figure 11:**
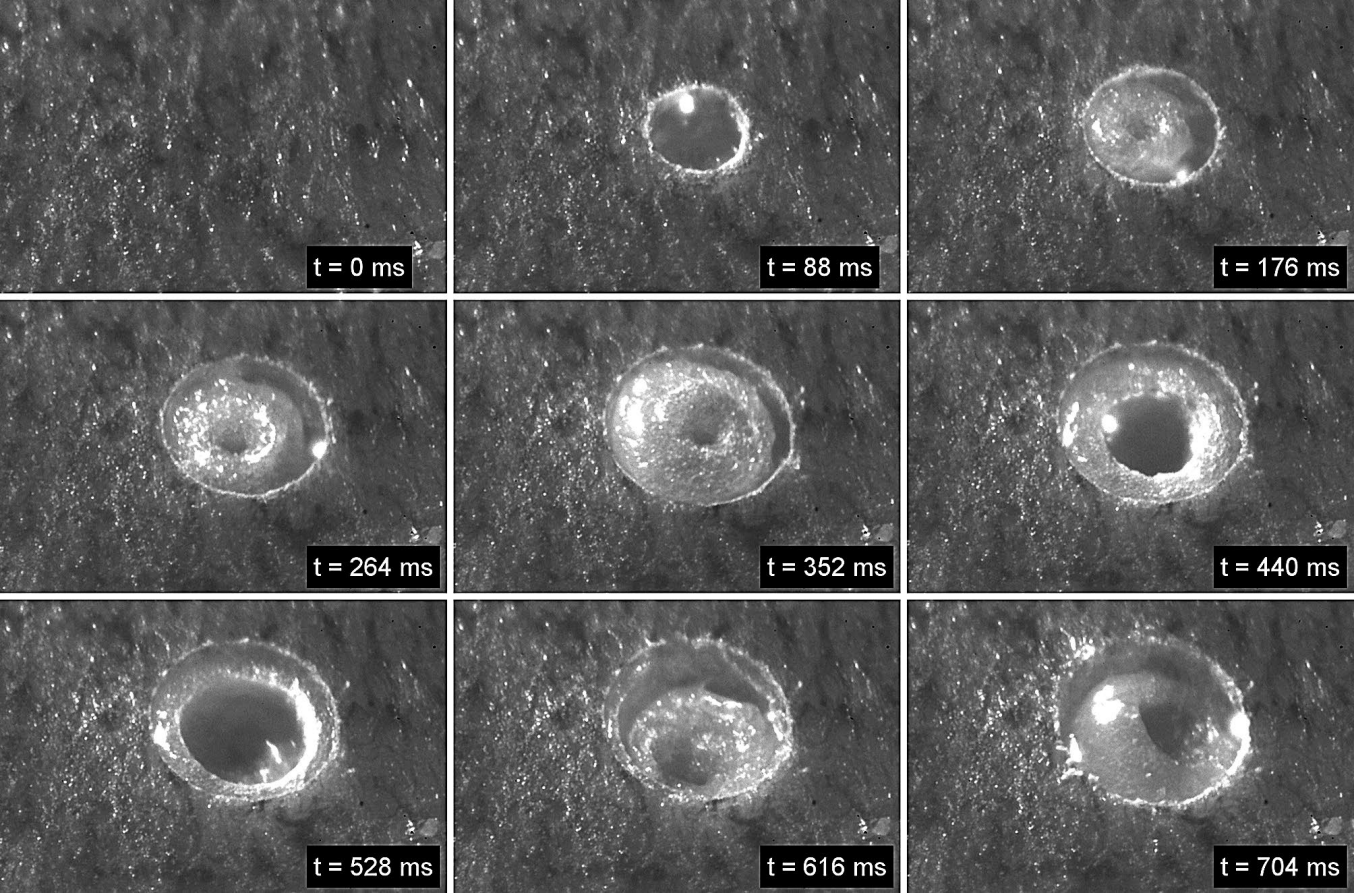
High speed imaging of laser ablation of healthy pig intestine at a repetition rate of 20 kHz and a spot separation of 100% between successive laser pulses. The diameter of the ablated region is 600 µm. The laser causes significant tissue coagulation as consequence of the cavitation effect and bubble formation.

**Figure 12:**
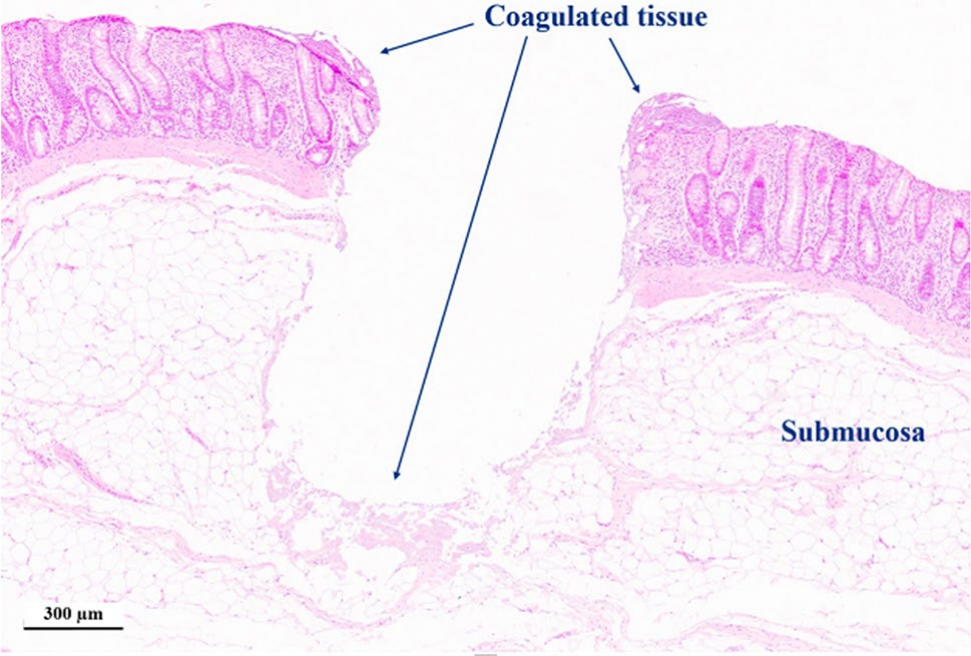
(**a**) Histology section after laser ablation of healthy pig intestine at a repetition rate of 20 kHz and a spot separation of 10% between successive laser pulses. Four repetitions of the spiral scan enable to remove the majority of the coagulated tissue caused by the cavitation effect.

Figure 11 and supporting material 2 show the high-speed footage for the laser ablation of healthy pig intestine at a repetition rate of 20 kHz and a low spot separation of 10% of the focused spot diameter. The adverse impact of the cavitation effect on the process is clearly visible including the generation and collapse of larger gas bubbles. The laser beam has been focused ~ 400 µm below the tissue surface to further de-tune the process. The outcome of the laser-tissue interaction is a mass of coagulated viscous tissue. Nevertheless, by using multiple iterations of the spiral laser scan using the same process parameters, a removal of the majority of the coagulated tissue can be achieved with a residual collateral thermal damage up to ~ 300 µm (Fig. 12). Beside the pulse energy of the laser and the spot separation, the impairment by the cavitation effect also depends on the tissue morphology and the mechanical properties of the individual tissue layers. The comparatively thick layer of the submucosa (Fig. 12) for the pig intestine containing predominately fatty tissue exhibited a more significant bubble formation and generation of coagulated viscous tissue for a low spot separation of 10% compared to the cancerous mouse intestine without a noticeable submucosa (see Fig. 9). A spot separation of 100% between successive laser pulses enabled in both cases to avoid the adverse impact of the cavitation on the tissue ablation.

Our findings suggest that picosecond pulsed laser ablation potentially has a role in the treatment of colonic neoplasms and therefore opens up a novel route to overcome existing limitations of surgical procedures when using electrocautery tools. Based on novel fibre delivery^18,19^, the process could be deployed through the working channel of a standard colonoscope. As common to all endoscopic therapeutic modalities, a biopsy would still be required for the histological diagnosis and accurate cancer staging, e.g. in combination with endoluminal ultrasound, prior to the laser ablation.

Despite the very inhomogeneous tissue morphology of both neoplastic and healthy colonic tissue, the laser enables to ablate well-defined cavities with a controllable depth (see Figs. 3, 4 and 9). Further in-vivo recovery experiments will be needed to confirm any benefit in terms of reduction in colonic perforation rate as a major complication during bowel surgery. The histopathological analysis demonstrated that the residual collateral damage is only minimal (see Figs. 7 and 9), i.e. smaller than 50 µm, which is a significant improvement compared to electrocautery tools. A tissue removal rate of 6.3 mm^3^/min has been achieved using a laser repetition rate of 20 kHz and a pulse energy of 85 µJ. This is a factor of 3 higher than reported for soft tissue ablation by means of the so-called ablation-cooling using of ultrashort laser pulses in a burst mode^15^. A further increase of the tissue removal rate can be achieved at higher laser repetition rates. However, this would impede the mechanical requirements for a miniature laser scanning device in order to utilise this technique at the distal end of an optical fibre for an endoscopic application in a clinical environment. At high laser repetition rates a separation of 100% of the spot diameter between successive laser pulses is crucial in order to avoid the adverse impact of the dynamics of the cavitation bubbles on the process. High-speed imaging of the tissue ablation did not reveal any observable ejection of tissue that might consist of live cells. This gives confidence that there would be no risk of seed-infecting other parts of the colon.

## Supplementary information

Supporting Material 1

Supporting Material 2

## Data Availability

All data are available from the Heriot-Watt University repository 10.17861/28e55003-0ef1-4c33-b53c-8470c50e8c6e.

## References

[1] Pitari GM (2003). Bacterial enterotoxins are associated with resistance to colon cancer. Proc. Natl. Acad. Sci..

[2] Fankhauser, F. & Kwasniewska, S. Lasers in ophthalmology: Basic, diagnostic, and surgical aspects: A review. (2003).

[3] Tanzi EL, Lupton JR, Alster TS (2003). Lasers in dermatology: Four decades of progress. J. Am. Acad. Dermatol..

[4] Newman J, Anand V (2002). Applications of the diode laser in otolaryngology. Ear Nose Throat J..

[5] Ossoff RH (1994). Clinical applications of lasers in otolaryngology—head and neck surgery. Lasers Surg. Med..

[6] Zhao W, Wang L (2018). Microdrilling of through-holes in flexible printed circuits using picosecond ultrashort pulse laser. Polymers.

[7] Vogel A, Venugopalan V (2003). Mechanisms of pulsed laser ablation of biological tissues. Chem. Rev..

[8] Vogel A, Noack J, Hüttman G, Paltauf G (2005). Mechanisms of femtosecond laser nanosurgery of cells and tissues. Appl. Phys. B.

[9] Tinne N (2016). Chromatically Encoded High-Speed Photography of Cavitation Bubble Dynamics Inside Inhomogeneous Ophthalmic Tissue.

[10] Linz N, Freidank S, Liang X-X, Vogel A (2016). Wavelength dependence of femtosecond laser-induced breakdown in water and implications for laser surgery. Phys. Rev. B.

[11] Ratkay-Traub I (2001). Ultra-short pulse (femtosecond) laser surgery: Initial use in LASIK flap creation. Ophthalmol. Clin. N. Am..

[12] Chen H, Li H, Sun YC, Wang Y, Lu PJ (2016). Femtosecond laser for cavity preparation in enamel and dentin: Ablation efficiency related factors. Sci. Rep..

[13] Hoy CL (2014). Clinical ultrafast laser surgery: Recent advances and future directions. Select. Top. Quant. Electron. IEEE J..

[14] Lanvin T (2015). Subsurface ablation of atherosclerotic plaque using ultrafast laser pulses. Biomed. Opt. Exp..

[15] Kerse C (2016). Ablation-cooled material removal with ultrafast bursts of pulses. Nature.

[16] Ancona A (2008). High speed laser drilling of metals using a high repetition rate, high average power ultrafast fiber CPA system. Opt. Express.

[17] Hashida M (2002). Ablation threshold dependence on pulse duration for copper. Appl. Surf. Sci..

[18] Jaworski P (2013). Picosecond and nanosecond pulse delivery through a hollow-core negative curvature fiber for micro-machining applications. Opt. Express.

[19] Mohanan SMPC (2019). Preclinical evaluation of porcine colon resection using hollow core negative curvature fibre delivered ultrafast laser pulses. J. Biophoton..

[20] Bidare P, Bitharas I, Ward RM, Attallah MM, Moore AJ (2018). Fluid and particle dynamics in laser powder bed fusion. Acta Mater..

[21] Ziegler A, Gonzalez L, Blikslager A (2016). Large animal models: The key to translational discovery in digestive disease research. Cell. Mol. Gastroenterol. Hepatol..

[22] Moser AR (1995). ApcMin: A mouse model for intestinal and mammary tumorigenesis. Eur. J. Cancer.

[23] Leclerc D, Deng L, Trasler J, Rozen R (2004). ApcMin/+ mouse model of colon cancer: Gene expression profiling in tumors. J. Cell. Biochem..

[24] Vogel, A., Linz, N. & Freidank, S. Method for laser machining transparent materials (Patent US8350183B2, 2013).

